# Caregiver Support Access and the Information Landscape: A Study of Information Sources and Formal Support Use

**DOI:** 10.1093/geroni/igaf122.4392

**Published:** 2025-12-31

**Authors:** Joseph Svec, Jun Chu, Eunbea Kim, In Jeong Hwang

**Affiliations:** St. Joseph’s University, Patchogue, New York, United States; University of Maryland, Baltimore County, Baltimore, Maryland, United States; Auburn University, Auburn, Alabama, United States; Iowa State University, Ames, Iowa, United States

## Abstract

The gap between the need for respite services and caregiver abilities to access them is well documented. (AARP, 2024). One reason for this gap is that many caregivers lack information regarding the cost and availability of respite services (Castro et al., 2023). Previous studies found that getting information on respite services can be cumbersome and the accessed information may be incomplete. (Leocadie et al., 2018). Drawing on institutional models of organizational behavior, we distinguish among three major types of information contexts: 1) Individualized, 2) Community-Level, and 3) Public-Level. The objective of our study is to examine the impact of information gathering on utilization of respite care among caregivers. Using data from the National Study of Caregivers (NSOC), we found that caregivers who seek information in the individual sphere have 16% higher odds (OR = 1.16) of using respite services whereas those who received information from community (OR = 0.82) or public (OR = 0.86) spheres have between 18% and 14% lower odds of using respite services. Rather, caregivers relying on community and public sphere for information had higher odds of receiving caregiving training and utilizing support group services. The results suggest that while individuals are more likely to seek functional, and costly, support via respite, public information sources are more likely to direct caregivers toward individualized self-help approaches via training to enhance caregiving activities or support group coping. These findings suggest that caregiver support policies should account for how informational disparities can create qualitative differences in formal support use.

